# Identifying Key Hematological and Biochemical Indicators of Disease Severity in COVID-19 and Non-COVID-19 Patients

**DOI:** 10.3390/diagnostics15111374

**Published:** 2025-05-29

**Authors:** Soo-Kyung Kim, Daewoo Pak, Jong-Han Lee, Sook Won Ryu

**Affiliations:** 1 Department of Laboratory Medicine, Ewha Womans University College of Medicine, Seoul 07985, Republic of Korea; skkim1@ewha.ac.kr; 2 Division of Data Science, College of Software Digital Healthcare Convergence, Yonsei University, Wonju 26493, Republic of Korea; dpak@yonsei.ac.kr; 3 Department of Laboratory Medicine, Wonju College of Medicine, Yonsei University, Wonju 26426, Republic of Korea; 4 Department of Laboratory Medicine, Kangwon National University School of Medicine, Chuncheon 24341, Republic of Korea

**Keywords:** COVID-19, hematologic biomarker, severity, cell population data, prognosis

## Abstract

**Background:** This study investigated hematological and biochemical parameters, including cell population data (CPD), to evaluate their association with severity in COVID-19 and non-COVID-19 patients. Identifying these parameters could aid in disease monitoring and clinical decision-making. **Methods**: A retrospective analysis of 8401 patients, including 603 COVID-19 cases and 7546 non-COVID-19 cases, were conducted. Complete blood count (CBC) and routine chemistry results obtained near the time of real-time polymerase chain reaction testing were analyzed to assess their associations with disease severity. A matched cohort analysis was performed to adjust for potential confounding factors, such as age and sex. **Results**: COVID-19 patients with elevated neutrophil side fluorescence light (NE-SFL), platelet-to-lymphocyte ratio (PLR), glucose, and aspartate aminotransferase (AST), along with decreased plateletcrit, were more likely to experience severe outcomes, such as hospitalization or death. In addition, decreased hemoglobin, lymphocyte side scatter (LY-SSC), and albumin, as well as increased leukocyte and monocyte side scatter (MO-SSC), were associated with a greater severity, regardless of COVID-19 status. **Conclusions**: We identified hematologic and chemical assay biomarkers that correlate with severe COVID-19. These findings may provide important information regarding the disease progression and clinical management. Incorporating these biomarkers into clinical decision support systems could facilitate personalized treatment strategies, optimize resource allocation, and enable real-time severity stratification.

## 1. Introduction

COVID-19 has a wide spectrum of clinical symptoms, ranging from mild symptoms to cytokine release syndrome and death [1]. Early prediction of disease severity is crucial for improving prognosis and optimizing healthcare resource allocation.

Laboratory assays, including complete blood count (CBC) and routine chemistry tests, are widely used during hospital stays or outpatient visits. Most clinical laboratories are equipped with high-throughput hematology analyzers, some of which are capable of differentiating leukocyte subsets by analyzing scatter and fluorescence properties, such as cell size, complexity, and DNA/RNA content, which are reported as research parameters in cell population data (CPD) [2,3]. There have been publications on some CPD parameters in COVID-19. However, these studies often focus on a limited range of leukocyte parameters, involve small patient cohorts, or lack healthy control groups [4,5,6].

The aim of this study is to evaluate various laboratory parameters including CBC, CPD, and routine chemistry assays, and to identify which parameters are associated with the severity of COVID-19. This study was performed on a large patient cohort using rigorous statistical methodologies to enhance reliability and validity.

## 2. Materials and Methods

### 2.1. Patient Population and Data Collection

We retrospectively reviewed the electronic medical records of all patients who visited Kangwon National University Hospital and underwent COVID-19 real-time polymerase chain reaction (RT-PCR) testing and a CBC between December 2020 and March 2023 (*n* = 8401).

**Figure 1:**
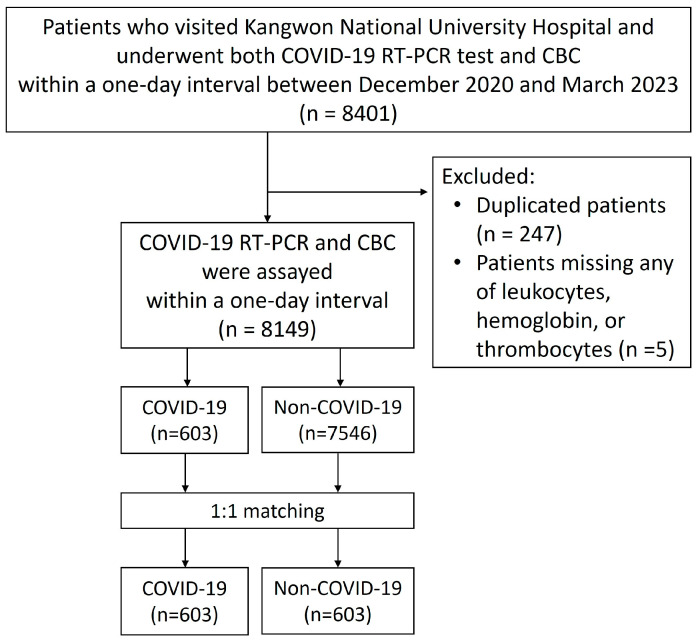
Inclusion and data analysis of this study. Abbreviation: CBC, complete blood count; RT-PCR, real-time polymerase chain reaction.

For COVID-19 positive patients, only CBC results obtained on the same day as the first positive COVID-19 RT-PCR test were included in the analysis. Patients who tested negative for COVID-19 RT-PCR throughout the study period were classified as the non-COVID-19 group, and their CBC results from the same day as the RT-PCR test were analyzed. If a patient had multiple CBC tests performed (*n* = 247), only one result was included in the analysis. If a same-day CBC was unavailable, results obtained within one day before or after the RT-PCR test were accepted. However, CBC results obtained more than one day apart from the RT-PCR test were excluded. Additionally, patients with missing data for leukocyte count, hemoglobin levels, or platelet count were excluded from the analysis (*n* = 5) (Figure 1).

Data were collected from electronic medical records. Patient outcomes were categorized into three groups: (1) outpatient care, (2) hospitalization including emergency room visits or intensive care unit (ICU) treatment, and (3) death.

### 2.2. Hematologic and Biochemical Assays

Hematologic analyses were performed using the Sysmex XN-10 (Sysmex, Kobe, Japan), and its CPD parameters were included in the analysis (Table 1) [7]. The neutrophil-to-lymphocyte ratio (NLR), platelet-to-lymphocyte ratio (PLR), and monocyte-to-lymphocyte ratio (MLR) were calculated.

Chemical assays were performed using the Hitachi LABOSPECT 008AS (Hitachi High-Technologies Corporation, Tokyo, Japan). COVID-19 RT-PCR tests were conducted using the PaxView^®^ SARS-CoV-2 real-time RT-PCR Kit (PaxGenBio, Anyang, Republic of Korea), the STANDARD^TM^ M nCoV Real-Time Detection kit (SD BIOSENSOR, Suwon, Republic of Korea), and the Real-Q Direct SARS-CoV-2 Detection Kit (BioSewoom, Seoul, Republic of Korea).

### 2.3. Statistical Analysis

Continuous variables were compared between COVID-19 and non-COVID-19 patients using the Wilcoxon rank-sum test, and categorical variables were compared using the chi-squared test. Additionally, descriptive analysis was performed across different patient groups, categorized as outpatients, hospitalized patients, and deceased patients.

To minimize potential bias from differences in age and sex distributions between the COVID-19 and non-COVID-19 patients, a cohort of non-COVID-19 patients was matched in a 1:1 ratio with COVID-19 patients based on age and sex. Patients with more than 50 missing values and variables with more than 20% missing data were excluded from the matching process. Missing values were imputed using K-Nearest Neighbors algorithm. The differences between the two patient groups were tested using the chi-square test for the categorical variables and the Wilcoxon signed-rank test for the continuous variables.

Upon analysis of baseline characteristics, we observed significant differences between COVID-19 positive (*n* = 603) and non-COVID-19 patients (*n* = 7546). The COVID-19 group demonstrated a significantly higher mean age than the non-COVID-19 group (66.8 ± 22.2 years vs. 55.9 ± 26.6 years, *p* < 0.001). Regarding sex distribution, although the proportion of males was slightly elevated in the COVID-19 group compared to the non-COVID-19 group, this difference was not statistically significant (52.1% vs. 49.4%, *p* = 0.216). Therefore, the effect of COVID-19 on the severity level were analyzed with the cumulative link mixed model using 1:1 matched dataset. The association between the severity level and patients’ routine test results at the time of COVID-19 diagnosis was evaluated separately for COVID-19 and non-COVID-19 patients using cumulative link models. Age, sex, and other variables with p<0.2 in the univariate analysis were included in the initial model, and backward elimination was applied until only variables with p<0.05 remained in the final model. Throughout this paper, the patient outcomes were coded as follows: 1 for outpatient care, 2 for hospitalization, and 3 for death. Then, a regression model for cumulative logits is defined as follows:

(1) $$log\frac{P\left(Y_{i}\leq j\right)}{P\left(Y_{i}>j\right)}=\theta_{0j}+x^{'}\beta ,j=1,2$$

where j refers to the j-th category of patient outcomes, x is a vector of explanatory variables, and $\theta_{0j}$ and β are the intercept for the cumulative logit for j and a vector of regression parameters, respectively.

Statistical analyses were performed using SPSS (version 28.0; IBM Corp., Armonk, NY, USA) and R (version 4.4.1; R Foundation for Statistical Computing, Vienna, Austria).

## 3. Results

### 3.1. Differences in Patient Characteristics Between COVID-19 and Non-COVID-19 Groups

Among 8149 patients (603 COVID-19 and 7546 non-COVID-19 patients), a 1:1 matched analysis was performed on 603 COVID-19 and 603 non-COVID-19 patients across 57 variables. The distributions of demographic characteristics and laboratory test results for each cohort are presented in Table 2.

### 3.2. Effect of COVID-19 on Severity Level

Among COVID-19 patients, 60 (10%) were outpatients, 439 (77%) were hospitalized, and 104 (17%) died. Among non-COVID-19 patients, 78 (13%) were outpatients, 467 (77%) were hospitalized, and 58 (10%) died.

The severe patient outcome was positively associated with COVID-19 (estimate = −0.522, *p* < 0.001), male (estimate = −0.309, *p* = 0.035), and older age (estimate = −0.018, *p* < 0.001).

### 3.3. Laboratory Factors Associated with COVID-19 Severity

Among COVID-19 patients, leukocyte count, neutrophil side-fluorescence light (NE-SFL) width, lymphocyte forward scatter (LY-FSC), LY-SFL width, monocyte side scatter (MO-SSC), PLR, glucose, and AST were positively correlated with severity. Conversely, hemoglobin, lymphocyte count, lymphocyte side scatter (LY-SSC), plateletcrit, and albumin were negatively correlated with severity (Table 3).

### 3.4. Laboratory Factors Associated with Non-COVID-19 Severity

Among non-COVID-19 patients, positively correlated factors with severity were leukocyte count, mean corpuscular volume (MCV), mean corpuscular hemoglobin concentration (MCHC), red cell distribution width-coefficient of variation (RDW-CV), neutrophil forward scatter (NE-FSC), neutrophil side scatter (NE-SSC) width, LY-FSC, LY-SFL width, LY-FSC width, MO-SSC, platelet large cell ratio, NLR, BUN, AST, and ALP.

Negatively correlated factors with severity were hemoglobin, mean corpuscular hemoglobin (MCH), eosinophils, LY-SSC, monocyte side-fluorescence light (MO-SFL), sodium, albumin, and creatinine (Table 4).

## 4. Discussion

In this study, we analyzed a large patient cohort and investigated an extensive range of hematological variables and clinical chemistry test parameters. We identified several key laboratory parameters significantly associated with COVID-19 severity.

Laboratory assays specific to COVID-19 severity, compared to non-COVID-19 patients, included elevated NE-SFL, increased lymphocyte count, decreased plateletcrit, increased PLR, increased glucose levels, and increased AST levels. Factors associated with worse outcomes in both COVID-19 and non-COVID-19 patients included decreased hemoglobin, increased leukocyte count, decreased LY-SSC, increased LY-FSC, widened LY-SFL, increased MO-SSC, decreased albumin levels, and elevated AST levels.

An increased leukocyte count was related with severity in both the COVID-19 and non-COVID-19 groups. However, studies report varying findings: some report that either an increased or decreased leukocyte count is associated with severity [8], while others suggest that leukocyte count is not correlated with COVID-19 severity [9]. The variation in leukocyte count depending on disease severity in COVID-19 can be explained as follows. Viral infection directly targets and destroys lymphocytes, leading to lymphopenia, which is further exacerbated in cases of cytokine storms. Both mechanisms contribute to leukopenia observed in severe COVID-19 cases. Conversely, immune system activation in response to infection can result in leukocytosis. In cases of severe immune overactivation or secondary bacterial infections, this increase in leukocyte count is more pronounced. Therefore, severe cases may exhibit either leukopenia due to immune suppression or leukocytosis driven by immune overactivation and secondary responses.

Changes in the numbers of neutrophils, eosinophils, lymphocytes, and monocytes have been reported to be associate with severe COVID-19 [2,8,9,10]. Additionally, severe COVID-19 is also associated with marked increase in immature neutrophils resulting from emergency myelopoiesis. In our study, elevated NE-SFL levels reflect increased nuclear heterogeneity and nucleic acid content in neutrophils, which is indicative of emergency myelopoiesis and the presence of immature or activated neutrophils. This phenomenon is commonly observed in severe COVID-19 as part of the cytokine storm and excessive innate immune activation [11].

Our analysis of lymphocyte-related variables revealed significant associations with severity in both the COVID-19 and non-COVID-19 groups. Decreased lymphocyte counts correlated with severe COVID-19 in our study, and it is well established that lymphopenia, particularly when lymphocyte counts fall below 1100/μL, is strongly correlated with poor prognosis and increased mortality rates in COVID-19 [12,13,14]. This lymphopenia is thought to result from the rapid depletion of peripheral T lymphocytes, including both CD4+ and CD8+ cells, during acute COVID-19, potentially due to their sequestration within target organs [15]. The exact mechanism underlying the marked lymphocyte decrease in severe COVID-19 is not yet fully understood, but several hypotheses have been proposed. Beyond lymphocyte infiltration and sequestration in the lungs, gastrointestinal tract, and lymphoid tissues [16], pro-inflammatory cytokines, including IL-6, are believed to contribute to lymphocyte depletion [17].

In addition, larger lymphocyte size (LY-FSC), reduced granules and vacuoles (LY-SSC), and a broader distribution of intracellular DNA content (LY-SFL width) were associated with poorer prognosis. Activated lymphocytes are typically larger, with increased granules and vacuoles with greater heterogeneity in DNA content. However, our study observed a paradoxical finding: larger lymphocyte size with reduced granules and vacuoles in severe COVID-19 cases. This may result from immune dysregulation rather than normal activation. Overstimulation followed by lymphocytes exhaustion can lead to enlarged lymphocytes that are functionally impaired, with a decline in granule and vacuole formation as their metabolic capacity diminishes. Additionally, SARS-CoV-2 may directly infect lymphocytes or disrupt their signaling pathways through interaction with the ACE2 receptor, potentially interfering with normal activation processes and causing aberrant morphological changes.

Regarding monocytes, increased MO-SSC was associated with COVID-19 severity, consistent with findings from previous studies [2]. Monocytes play critical roles in cytokines production, phagocytosis, and lymphocyte activation [18]. An increased MO-SSC indicates the presence of active monocytes with greater amounts of granules and vacuoles. Previous studies have reported that a decreased monocyte count is associated with COVID-19 related cytokine-storm syndrome and death [2,9]. This decrease may result from monocyte exhaustion due to extravasation and migration to affected tissues [19]. However, in this study, no correlation was found between a decreased monocyte count and COVID-19 severity. This discrepancy may be attributed to differences in the severity levels across studies. While our study used MO-SSC to indirectly measure monocyte activation, MO-SSC mainly shows cell shape changes rather than actual immune function. Recent studies have shown that monocyte HLA-DR expression is an important marker of immune system problems in severe inflammatory conditions, including COVID-19. Notably, HLA-DR expression on monocytes drops significantly in critically ill patients and returns to normal as patients improve and their lymphocyte counts recover [20,21]. The nuanced regulation of HLA-DR across classical and non-classical monocyte subpopulations, modulated by cytokines such as IL-10 and cortisol, illustrates the intricate nature of monocyte responses during severe infections [22]. Although our study only looked at cell scatter measurements, the increase in MO-SSC we observed in severe COVID-19 patients matches expected changes in monocyte activation and internal cell structure. Future research that includes detailed analysis of monocyte subtypes and measures HLA-DR levels could provide better understanding of immune suppression and help develop targeted treatments.

A decrease in plateletcrit and an increase in the PLR were found to correlate with severity, whereas platelet count and MPV did not. Platelets are important mediators of inflammation [23]. Platelets are usually inactive in the bloodstream, but they can rapidly activate in response to vascular injury, cytokines, or infection. This activation allows them to perform roles beyond hemostasis, including inflammation and immune regulation [24]. Additionally, cytokines may impair bone marrow function, disrupting megakaryocyte development and leading to thrombocytopenia [25]. The lungs also support platelet production, serving as a site for megakaryocyte migration and platelet generation [26]. Therefore, the decrease in plateletcrit observed in severe cases may reflect increased platelet consumption and impaired production due to inflammatory and bone marrow dysfunction. In contrast, the elevated PLR in critically ill patients is primarily driven by marked lymphopenia rather than thrombocytopenia [27]. One of the largest studies related to platelets, conducted by Barrett et al. on a cohort of 3915 COVID-19 patients, reported that decreased platelet counts, increased platelet size (MPV), and elevated immature platelet fraction (IPF) were significantly associated with critical illness and mortality [28]. Other studies have shown variability in platelet count findings, with some reporting a decrease and others finding no significant impact [9,27,29]. IPF was excluded from our analysis because it had more than 20% missing data. However, previous studies have reported that increased immature platelets are observed in severe COVID-19 [30,31]. Paradoxically, decreased plateletcrit was associated with increased severity in this study. This may reflect a bone marrow response to increased platelet consumption, but that the compensation is insufficient. Meanwhile, inflammation and coagulopathy contribute to lymphocyte depletion and platelet activation, further worsening disease prognosis. These processes create a vicious cycle of inflammation and coagulopathy. Therefore, using these test results could provide information on disease progression and outcomes.

Our analysis revealed routine chemistry assays, including renal and liver function test results, such as higher glucose, lower albumin, and higher AST levels, are associated with severe COVID-19. Hyperglycemia impairs innate immunity, promotes oxidative stress and endothelial dysfunction, and may amplify the cytokine storm [32,33]. Additionally, glycosylation of ACE2 receptors under hyperglycemic conditions can enhance SARS-CoV-2 entry and disease severity [34]. A decreased albumin level, known for its anti-inflammatory and antioxidant properties, has been associated with poor outcomes in severe COVID-19, likely reflecting systemic inflammation and nutritional status. Elevated AST levels in severe COVID-19 may reflect multi-organ involvement, including hepatocellular injury, muscle breakdown, or cardiac inflammation. Elevated BUN and ALP levels indicate renal and hepatic dysfunction, both of which are common in severe cases [35,36]. These results highlight systemic inflammation and organ dysfunction, supporting severity stratification and guiding treatment.

Recent studies have further confirmed the predictive value of blood test results in COVID-19. Several research groups have shown that measurements like NLR, PLR, and liver enzymes (AST, ALT) help identify high-risk pediatric COVID-19 patients early [37,38]. These findings match our results and support the usefulness of cell measurements—particularly NE-SFL, LY-SSC, and MO-SSC—in showing how severe the disease is. Additionally, broader public health studies have shown the importance of combining laboratory testing with location-based and behavioral approaches for better community disease control [39]. These different viewpoints strengthen how our findings can be used in clinical settings and applied more widely, especially when making decisions about resources during pandemics.

This study has several limitations. First, it is an observational and retrospective study, which limits the ability to establish causality. The patient population was restricted to individuals tested and treated within specific healthcare settings. Future studies involving larger and more diverse populations are needed.

Additionally, the analysis did not account for potential confounding variables such as comorbidities, complications, or treatment variations. As the primary objective of this study was to evaluate the predictive value of laboratory parameters for disease severity, these factors were not incorporated into our statistical modeling. However, comorbidities are known to significantly influence COVID-19 outcomes [40,41,42]. Cardiovascular disease, cancer, diabetes, obesity, and immunodeficiency have been reported to be associated with increased severity [41,42]. To assess their potential impact within our cohort of 8149 patients, we calculated the prevalence of selected comorbidities and complications. Specifically, we identified 88 patients with chronic renal disease, 53 with cirrhosis, 25 with sepsis, and 1 with myocarditis—together representing approximately 2.0% of the total cohort. Although we did not perform comorbidity-adjusted analyses, the relatively low prevalence of these conditions suggests a limited impact on the overall associations observed. Nevertheless, we recognize this as a meaningful limitation and have discussed it accordingly.

Furthermore, complete records of treatments given to patients were not consistently available throughout our study group. As a result, we could not properly evaluate how different treatments might have affected patient outcomes. This limitation should be kept in mind when considering our findings, since various therapies could have changed how patients progressed, regardless of their initial condition and laboratory test results. Future studies should carefully document all treatments to better understand how they might influence the relationship between laboratory markers and disease outcomes.

Lastly, our use of outcome-based categories (outpatient care, hospitalization, and death) as surrogates for disease severity represents a methodological compromise. This approach was necessitated by the retrospective design of our study and the limited availability of comprehensive clinical data including oxygen saturation measurements, respiratory rates, and radiographic findings across our cohort. While this stratification system effectively reflects real-world clinical progression and medical resource allocation patterns, it does not precisely align with internationally standardized severity definitions established by NIH and WHO, which classify COVID-19 as mild, moderate, severe, or critical based on specific physiological parameters [43,44]. Consequently, although our classification system captures important clinical outcomes as they occur in actual practice, it may not provide the detailed diagnostic precision needed for ideal comparison across different countries. Upcoming prospective investigations with systematic and comprehensive data collection protocols should endeavor to incorporate these standardized criteria to achieve more precise severity classification and enhance the interpretability of findings across different healthcare contexts.

Investigating the potential role of these parameters in predicting outcomes and guiding treatment strategies could provide important information to improve the clinical management of COVID-19 patients. Integrating these laboratory parameters into clinical decision support systems could improve their clinical utility by allowing real-time severity assessment and personalized treatment strategies [45].

## 5. Conclusions

We identified various laboratory parameters associated with the severity of COVID-19, providing potential biomarkers for assessing disease severity and informing clinical decision-making. Further research is needed to expand on these findings and deepen our understanding of the complex interplay of factors that influence COVID-19 severity.

## Figures and Tables

**Table 1:** Cell population data parameters included in this study.

Parameters	Alternative Name	Description	Meaning
MicroR		Micro RBC ratio	Microcytic population of red blood cells
MacroR		Macro RBC ratio	Macrocytic population of red blood cells
NE-FSC	NE-Z	Neutrophils cell size	Changes may reflect abnormal neutrophil size
NE-SFL	NEUT-RI, NE-Y	Neutrophils reactivity index. Metabolic activity	Increases with higher nucleic acid content, indicating immature or reactive neutrophils
NE-SSC	NEUT-GI, NE-X	Neutrophils granularity index	Increases with greater amounts of granules, vacuoles and other cytoplasmic inclusions
NE-FSC width	NE-WZ	Neutrophils cell size and the width of dispersion	Reflects neutrophil size heterogeneity
NE-SFL width	NE-WY	Neutrophils fluorescence intensity and the width of dispersion	Indicates heterogeneity in nucleic acid content among neutrophils
NE-SSC width	NE-WX	Neutrophils complexity and width of dispersion of the events measured	Indicates heterogeneity in granularity among neutrophils
LY-FSC	LY-Z	Lymphocytes cell size	Its change reflects presence of abnormal sized cells (e.g., increase with activated lymphocyte or decrease with pyknotic lymphocytes, etc.)
LY-SFL	LY-Y	Lymphocytes metabolic activity and/or permeability of the cell membrane. Lymphocytes fluorescence intensity	Increase in proportion to the amount of nucleic acid, such as in activated or abnormal lymphocytes and blast cells, etc.
LY-SSC	LY-X	Lymphocytes cell complexity	Increase in the presence of greater amounts of granules or vacuoles (e.g., large granular lymphocyte)
LY-FSC width	LY-WZ	Lymphocytes cell size and the width of dispersion	Reflects lymphocyte size heterogeneity
LY-SFL width	LY-WY	Lymphocytes fluorescence intensity and the width of dispersion	Indicates heterogeneity in nucleic acid content among lymphocytes
LY-SSC width	LY-WX	Lymphocytes complexity and width of dispersion of the events measured	Indicates heterogeneity in granularity among lymphocytes
HFLC		High fluorescence lymphocyte count	It represents activated cells (antibody-secreting B lymphocytes and plasma cells) if systemic hematological diseases can be excluded
MO-FSC	MO-Z	Monocytes cell size	Its change reflects presence of abnormal sized cells
MO-SFL	MO-Y	Monocytes metabolic activity and/or permeability of cell membrane. Monocytes fluorescence intensity	Increase in proportion to the amount of cellular nucleic acid (e.g., activated monocytes and monoblasts
MO-SSC	MO-X	Monocytes cells complexity	Increase in the presence of greater amounts of granules, vacuoles and other cytoplasmic inclusions. Decrease in the presence of a lower cell complexity
MO-FSC width	MO-WZ	Monocytes cell size and the width of dispersion	Reflects monocyte size heterogeneity
MO-SFL width	MO-WY	Monocytes fluorescence intensity and the width of dispersion	Indicates heterogeneity in nucleic acid content among monocytes
MO-SSC width	MO-WX	Monocytes complexity and width of dispersion of the events measured	Indicates heterogeneity in granularity among monocytes
P-LCR		Platelet large cell ratio	Its increase suggests possible risk of thrombosis
PDW		Platelet distribution width	Its increase reflects size variation. Its increase may be associated with vascular disease or certain cancers

Abbreviations: HFLC, high fluorescence lymphocytes; LY, lymphocyte; LY-FSC, lymphocyte forward scatter; LY-SFL, lymphocyte side-fluorescence light; LY-SSC, lymphocyte side scatter; MacroR, macrocytic red blood cells; MicroR, microcytic red blood cells; MO, monocyte; MO-FSC, monocyte forward scatter; MO-SFL, monocyte side-fluorescence light; MO-SSC, monocyte side scatter; NE, neutrophil; NE-FSC, neutrophil forward scatter; NE-SFL, neutrophil side-fluorescence light; NE-SSC, neutrophil side scatter; NEUT-GI, neutrophil granulocyte immature; NEUT-RI, neutrophil regenerative immature; PDW, platelet distribution width; P-LCR, platelet large cell ratio; RBC, red blood cell.

**Table 2:** Demographic and laboratory test results of 1:1 matched COVID-19 and non-COVID-19 groups.

	COVID-19	Non-COVID-19	*p*-Value
	Mean (SD)	Mean (SD)	
Age	66.8 (22.2)	66.8 (22.2)	0.775
Sex, male *	314 (52.1%)	314 (52.1%)	1.000
CBC			
Hemoglobin, g/dL	11.2 (2.2)	11.9 (2.2)	<0.001
Erythrocytes, ×10^12^/L	3.7 (0.8)	4.0 (0.8)	<0.001
Leukocytes, ×10^9^/L	8.6 (5.8)	8.6 (9.8)	0.970
Platelets, ×10^9^/L	224.7 (104.1)	243.9 (99.5)	0.001
Hematocrit	34 (6.3)	36 (6.2)	<0.001
Erythrocytes			
MCV	91.6 (6.3)	91.7 (6.6)	0.561
MCH	30.1 (2.4)	30.4 (2.5)	0.042
MCHC	32.9 (1.4)	33.1 (1.3)	0.002
nRBC (>0) *	73 (12.1%)	134 (22.2%)	<0.001
MicroR	2.5 (4.2)	2.4 (4.4)	0.030
MacroR	4.7 (2.8)	4.6 (2.8)	0.673
RDW-CV	14.3 (2.6)	14.0 (2.3)	0.004
RDW-SD	47.4 (8.3)	46.6 (7.6)	0.049
Granulocytes			
Neutrophils, ×10^9^/L	6.3 (4.8)	5.8 (5.8)	0.035
NE-FSC, ch	89.8 (5.0)	90.6 (4.6)	0.006
NE-SFL, ch	50.7 (6.3)	49.8 (4.0)	0.070
NE-SSC, ch	154.9 (5.2)	154.9 (4.5)	0.798
NE-SSC width, ch	313.1 (26.4)	306.8 (26.0)	<0.001
NE-SFL width, ch	650.2 (97.1)	632.5 (127.8)	<0.001
NE-FSC width, ch	750.1 (96.3)	738.5 (96.9)	0.060
Immature granulocytes, ×10^9^/L	0.1 (0.3)	0.1 (1.9)	<0.001
Basophils, ×10^9^/L	0 (0)	0 (0)	<0.001
Eosinophils, ×10^9^/L	0.1 (0.2)	0.2 (0.3)	<0.001
Lymphocytes			
Lymphocytes, ×10^9^/L	1.4 (2.7)	1.8 (1.1)	<0.001
LY-SSC, ch	78.8 (3.7)	79.0 (3.6)	0.246
LY-SFL, ch	69.4 (5.8)	70.6 (4.8)	<0.001
LY-FSC, ch	58.2 (2.8)	58.3 (2.1)	0.170
LY-SSC width, ch	582.4 (108.7)	553.3 (79.2)	<0.001
LY-SFL width, ch	900.7 (188.3)	880.5 (110.4)	0.179
LY-FSC width, ch	601.3 (137.5)	569.6 (90.9)	<0.001
HFLC, ×10^9^/L	0 (0.1)	0 (0)	<0.001
Monocytes			
Monocytes, ×10^9^/L	0.7 (0.4)	0.7 (1.4)	0.142
MO-SSC, ch	121.2 (3.3)	120.0 (3.1)	<0.001
MO-SFL, ch	114.6 (11.1)	116.1 (8.7)	0.017
MO-FSC, ch	66.8 (4.0)	67.4 (3.6)	0.005
MO-SSC width, ch	261.4 (37.3)	259.7 (27.6)	0.324
MO-SFL width, ch	709.0 (131.3)	685.9 (94.9)	<0.001
MO-FSC width, ch	672.3 (126)	668.5 (120.1)	0.376
TNC	8.6 (5.8)	8.6 (9.9)	0.988
Thrombocytes			
Plateletcrit	0.2 (0.1)	0.2 (0.1)	0.015
P-LCR	23.6 (8.6)	21.7 (7.3)	<0.001
MPV	9.9 (1.1)	9.6 (0.9)	<0.001
PDW	10.6 (2.5)	10.2 (1.9)	0.008
Ratios			
NLR	7.2 (9.1)	4.5 (6.3)	<0.001
PLR	222.2 (159.7)	174.3 (127.0)	<0.001
MLR	0.6 (0.6)	0.5 (0.7)	<0.001
Chemical assay			
Sodium	138.7 (4.4)	139.6 (3.7)	<0.001
Glucose	125.5 (46.7)	127.2 (54.6)	0.914
Albumin	3.3 (0.7)	3.6 (0.7)	<0.001
Renal function test			
BUN	20.8 (17.9)	18.7 (13.8)	0.039
Creatinine	1.1 (1.3)	1.0 (1.1)	0.060
Liver function tests			
AST	49.5 (165.7)	54.5 (271.5)	0.005
ALT	50.8 (315.2)	44.4 (254.4)	0.079
ALP	110.7 (91.6)	112.0 (99.3)	0.955
Total bilirubin	0.9 (1.1)	0.9 (1.5)	0.259

* Categorical variables are presented as a number (%). The meaning of the cell population data parameters can be found in Table 1. Abbreviations: ALP, alkaline phosphatase; ALT, alanine aminotransferase; AST, aspartate aminotransferase; BUN, blood urea nitrogen; CBC, complete blood count; HFLC, high fluorescence lymphocytes; LY-FSC, lymphocyte forward scatter; LY-SFL, lymphocyte side-fluorescence light; LY-SSC, lymphocyte side scatter; MacroR, macrocytic red blood cells; MCH, mean corpuscular hemoglobin; MCHC, mean corpuscular hemoglobin concentration; MCV, mean corpuscular volume; MicroR, microcytic red blood cells; MLR, monocyte-to-lymphocyte ratio; MO-FSC, monocyte forward scatter; MO-SFL, monocyte side-fluorescence light; MO-SSC, monocyte side scatter; MPV, mean platelet volume; NE-FSC, neutrophil forward scatter; NE-SFL, neutrophil side-fluorescence light; NE-SSC, neutrophil side scatter; NLR, neutrophil-to-lymphocyte ratio; nRBC, nucleated red blood cells; PDW, platelet distribution width; P-LCR, platelet large cell ratio; PLR, platelet-to-lymphocyte ratio; RDW-CV, red cell distribution width-coefficient of variation; RDW-SD, red cell distribution width-standard deviation; SD, standard deviation; TNC, total nucleated cells.

**Table 3:** Analysis results from the multivariable cumulative link model in the COVID-19 patients.

	Estimate	*p*-Value
Age	−0.1	0.421
Sex, male	−0.4	0.072
CBC		
Hemoglobin, g/dL	0.458	0.001
Leukocytes, ×10^9^/L	−0.442	0.001
Granulocytes		
NE-SFL width, ch	−0.261	0.028
Lymphocytes		
Lymphocytes, ×10^9^/L	0.278	0.038
LY-SSC, ch	0.407	0.008
LY-FSC, ch	−0.334	0.015
LY-SFL width, ch	−0.437	0.004
Monocytes		
MO-SSC, ch	−0.319	0.007
Thrombocytes		
Plateletcrit	0.286	0.021
Ratio		
PLR	−0.353	0.003
Chemical assay		
Glucose	−0.233	0.047
Albumin	0.832	<0.001
Liver function tests		
AST	−0.786	0.003

Abbreviations: AST, aspartate aminotransferase; CBC, complete blood count; LY-FSC, lymphocyte forward scatter; LY-SFL, lymphocyte side-fluorescence light; LY-SSC, lymphocyte side scatter; MO-SSC, monocyte side scatter; NE-SFL, neutrophil side-fluorescence light; PLR, platelet-to-lymphocyte ratio.

**Table 4:** Analysis results from the multivariable cumulative link model in the non-COVID-19 patients.

	Estimate	*p*-Value
Age	−0.214	<0.001
Sex, male	−0.5	<0.001
CBC		
Hemoglobin, g/dL	0.537	<0.001
Leukocytes, ×10^9^/L	−0.113	0.029
Erythrocytes		
MCV	−3.164	<0.001
MCH	3.653	<0.001
MCHC	−1.886	<0.001
RDW-CV	−0.292	<0.001
Granulocytes		
NE-FSC, ch	−0.161	<0.001
NE-SSC width, ch	−0.242	<0.001
Eosinophils, ×10^9^/L	0.142	<0.001
Lymphocytes		
Lymphocytes, ×10^9^/L		
LY-SSC, ch	0.263	<0.001
LY-FSC, ch	−0.28	<0.001
LY-SFL width, ch	−0.175	<0.001
LY-FSC width, ch	−0.143	0.003
Monocytes		
MO-SSC, ch	−0.438	<0.001
MO-SFL, ch	0.175	<0.001
MO-FSC width, ch	−0.099	0.029
Thrombocytes		
P-LCR	−0.095	0.003
Ratio		
NLR	−0.319	<0.001
Chemical assay		
Sodium	0.123	<0.001
Albumin	0.215	<0.001
Renal function test		
BUN	−0.156	0.001
Creatinine	0.166	<0.001
Liver function tests		
AST	−0.107	0.004
ALP	−0.235	<0.001

Abbreviations: ALP, alkaline phosphatase; AST, aspartate aminotransferase; BUN, blood urea nitrogen; CBC, complete blood count; LY-FSC, lymphocyte forward scatter; LY-SFL, lymphocyte side-fluorescence light; LY-SSC, lymphocyte side scatter; MCH, mean corpuscular hemoglobin; MCHC, mean corpuscular hemoglobin concentration; MCV, mean corpuscular volume; MO-FSC, monocyte forward scatter; MO-SFL, monocyte side-fluorescence light; MO-SSC, monocyte side scatter; NE-FSC, neutrophil forward scatter; NE-SSC, neutrophil side scatter; NLR, neutrophil-to-lymphocyte ratio; P-LCR, platelet large cell ratio; RDW-CV, red cell distribution width-coefficient of variation.

## Data Availability

The data presented in this study are available on request form the corresponding author.
